# Data on antiplatelet aggregation, anticoagulation and antioxidant activities of *Canna edulis* Ker rhizome and its secondary metabolites

**DOI:** 10.1016/j.dib.2020.106115

**Published:** 2020-08-01

**Authors:** Thi Minh Hang Nguyen, Hong Luyen Le, Quoc Hung Tran, Thi Thoa Ha, Bich Hau Bui, Nguyen Thanh Le, Van Hung Nguyen, Thi Van Anh Nguyen

**Affiliations:** a Center of Drug Research and Development, Institute of Marine Biochemistry, Vietnam Academy of Science and Technology, Hoang Quoc Viet, Cau Giay, Hanoi, Viet Nam; b Department of Life Sciences, University of Science and Technology of Hanoi, Vietnam Academy of Science and Technology, Hoang Quoc Viet, Cau Giay, Hanoi, Viet Nam; c Lam Dong Medical College, Da Lat, Vietnam

**Keywords:** *Canna edulis Ker*, Antiplatelet, Anticoagulant, Antioxidant, Flavonoids compounds

## Abstract

*Canna edulis* Ker rhizome has been used in Traditional Vietnamese Medicine to prevent and treat heart diseases without thorough scientific evidence. The data presented in this article characterize the antiplatelet aggregation, anticoagulant and antioxidant activity of *C. edulis* rhizome extracts and the bio-guided isolation of bioactive compounds from the active fraction. The data on tested bioactivities of isolated compounds were also provided. The inhibitory effect on adenosine diphosphate- and collagen-induced human platelet aggregation was evaluated through three parameters: percentage inhibition of platelet aggregation, aggregation velocity and area under the platelet aggregation curve. Prothrombin time, activated partial thromboplastine time and thrombine time were measured to examine the anticoagulant activity. The free radical scavenging ability was assessed with DPPH and ABTS assays. The structures of compounds were elucidated by NMR and MS spectroscopic methods. The data showed that the ethyl acetate fraction showed the most potent antiplatelet aggregation, anticoagulant and antioxidant activity. Seven known compounds: 5-hydroxy-6-methyl-2*H*-pyran-2-one **(1)**, epimedokoreanone A **(2)**, nepetoidin B **(3)**, ferulic acid **(4)**, caffeic acid **(5)**, hydroxytyrosol **(6)**, and 1H-indole-3-carboxaldehyde **(7)** were isolated from this active fraction. Moreover, this article provided experimental data on antiplatelet effect of epimedokoreanone A **(2)** and nepetoidine B **(3)**, anticoagulant and antioxidant activity of epimedokoreanone A **(2)** and also antiplatelet and antioxidant activity of 5-hydroxy-6-methyl-2*H*-pyran-2-one **(1)**.

**Specifications Table**

**Table:** 

Subject	Pharmacology
Specific subject area	Bioactivities: antiplatelet, anticoagulant, antioxidant
Type of data	Table, Figure
How data were acquired	Collection of plant, antiplatelet aggregation assay, coagulant assay, antioxidant assay NMR spectroscopy: Bruker AM500 FT-NMR ; APCI-MS : AGILENT 1200 series LC-MSD Ion Trap
Data format	Raw and analyzed
Parameters for data collection	Data were collected from experiments described in the methods section The isolated compounds were purified by column chromatography
Description of data collection	Data were collected from experiments described in the methods section The isolated compounds were identified by NMR and APCI-MS spectroscopy
Data source location	University of Science and Technology of Hanoi, Vietnam Academy of Science and Technology, Hanoi, Vietnam
Data accessibility	With the article
Subject	Pharmacology
Specific subject area	Bioactivities: antiplatelet, anticoagulant, antioxidant
Type of data	Table, Figure
How data were acquired	Collection of plant, antiplatelet aggregation assay, coagulant assay, antioxidant assay NMR spectroscopy: Bruker AM500 FT-NMR ; APCI-MS : AGILENT 1200 series LC-MSD Ion Trap
Data format	Raw and analyzed
Parameters for data collection	Data were collected from experiments described in the methods section The isolated compounds were purified by column chromatography
Description of data collection	Data were collected from experiments described in the methods section The isolated compounds were identified by NMR and APCI-MS spectroscopy
Data source location	University of Science and Technology of Hanoi, Vietnam Academy of Science and Technology, Hanoi, Vietnam
Data accessibility	With the article
Related research article	Nguyen T.M.H., Le H.L., Ha T.T., Bui B.H., Nguyen T.L., Nguyen, V.H., Nguyen T.V.A*. Inhibitory effect on human platelet aggregation and coagulation and antioxidant activity of *Canna edulis* Ker rhizome and its secondary metabolites. J Ethnopharmacol, in press.

Value of the data

• The data provide scientific evidences on antiplatelet aggregation, anticoagulant and antioxidant activity of *C. edulis* rhizome and its secondary metabolites.
• This article can help in further *in-vitro* and *in vivo* research to identify bioactive molecules with antiplatelet, anticoagulant and antioxidant activity from *C. edulis* rhizome, clarify mechanisms of action and develop new anti-thrombosis and antioxidant agents.
• The reported bioactivities of *C. edulis* rhizome and its secondary metabolite in this article also demonstrate the potential use of this edible plant to develop functional food for treatment and prevention of heart- and oxidative stress- related diseases.
• The provided information on the spectroscopic data of isolated compounds from *Canna edulis* could be useful for the analysis of spectra and determination of the structure of isolated compounds from other *Canna* species.
• This data can serve as benchmark for other researchers to elucidate the structures of caffeic derivatives.

## 1 Data description

**Fig. 1:**
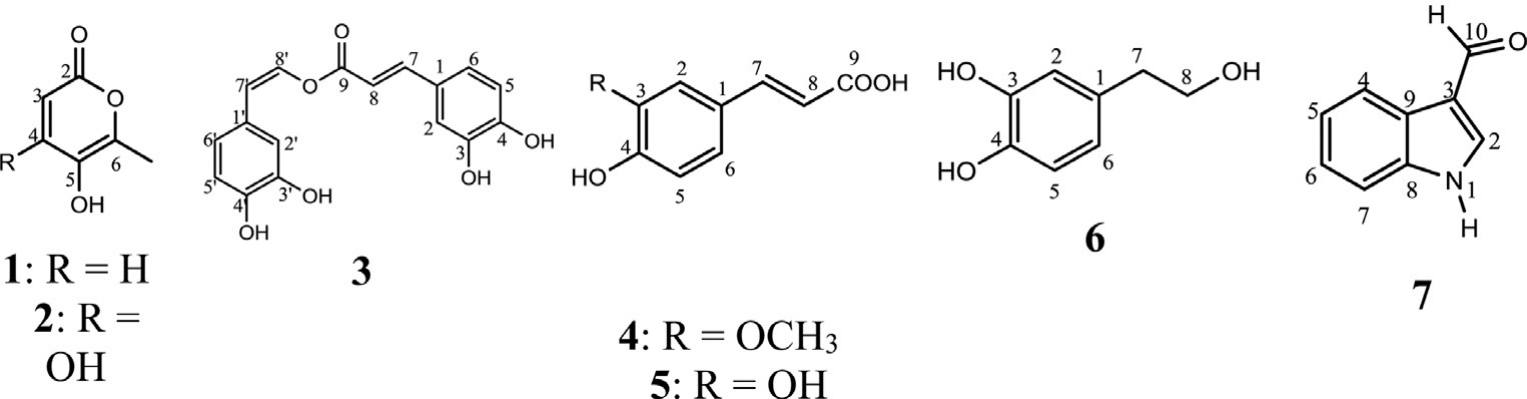
Structures of isolated compounds from the ethyl acetate fraction of *C. edulis* rhizome.

**Fig. 2:**
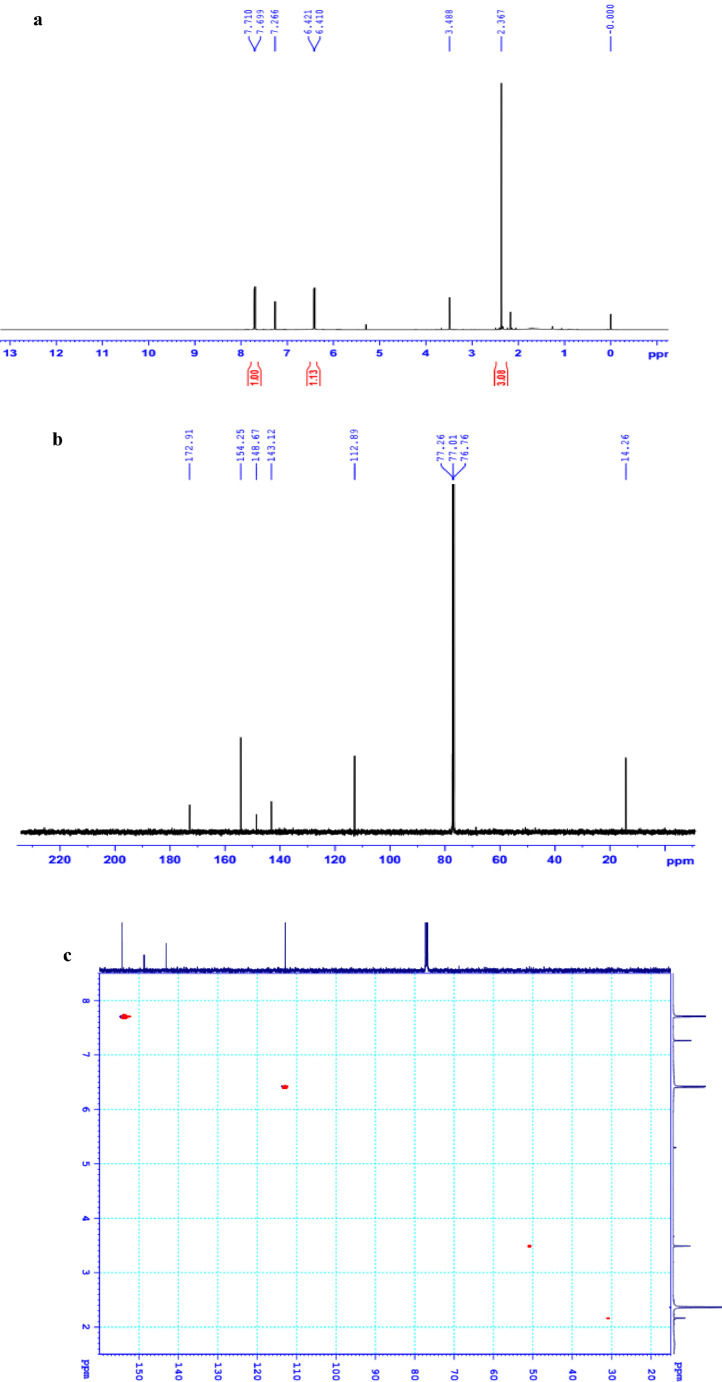

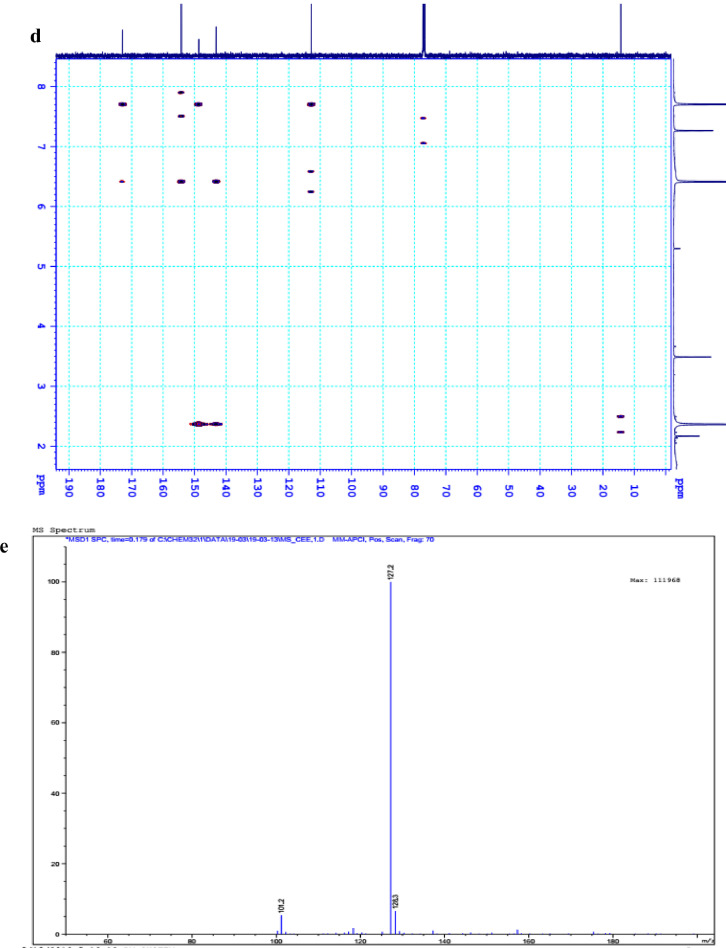
**a**. ^1^H NMR (500 MHz, CDCl_3_) of **1, b**. ^13^C NMR (125 MHz, CDCl_3_) of **1, c**. HSQC of **1, d**. HMBC of **1, e**. (+) APCI-MS of **1**.

**Fig. 3:**
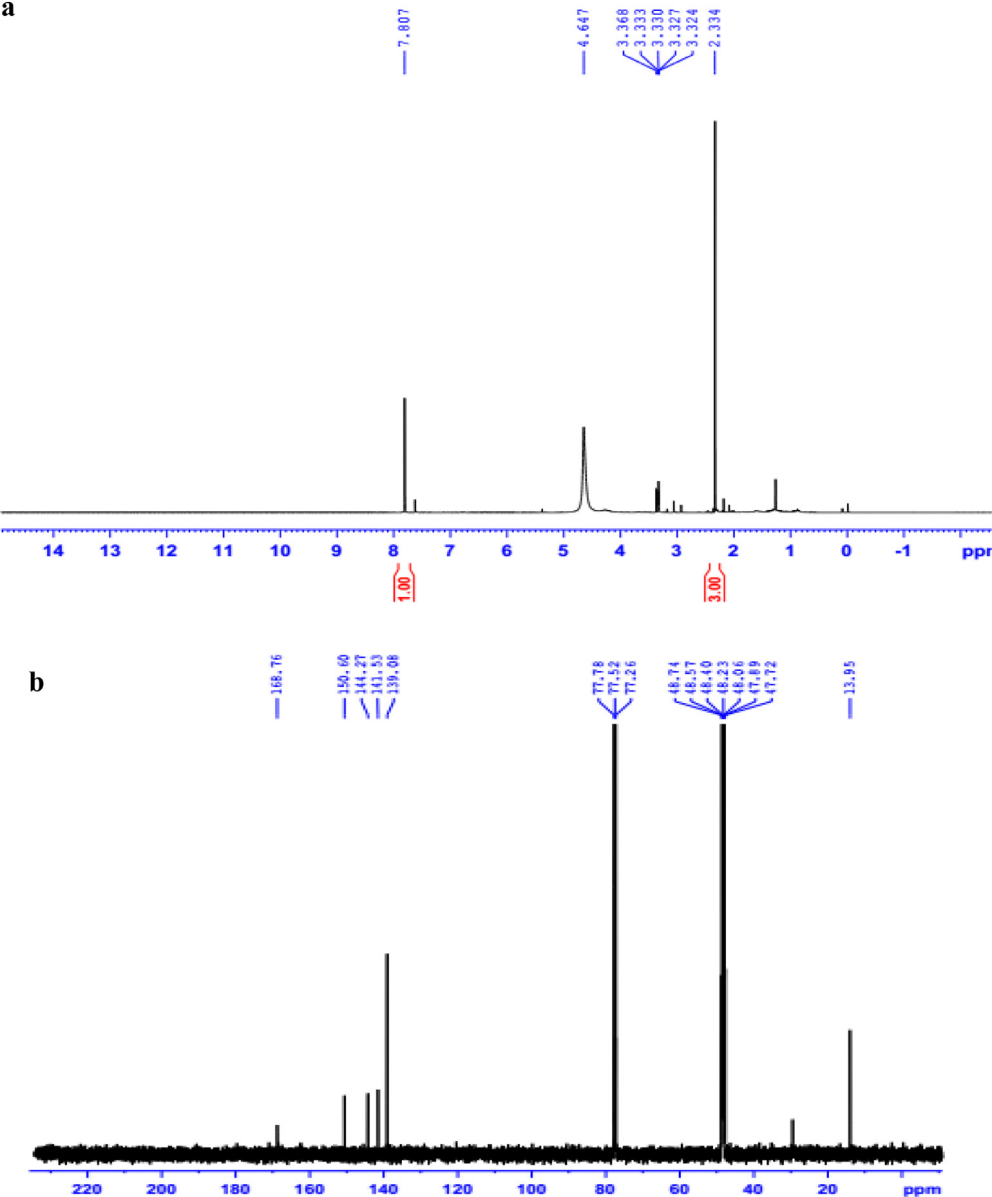
**a**. ^1^H NMR (500 MHz, CDCl_3_+CD_3_OD) of **2, b**. ^13^C NMR (125 MHz, CDCl_3_+CD_3_OD) of **2.**

**Fig. 4:**
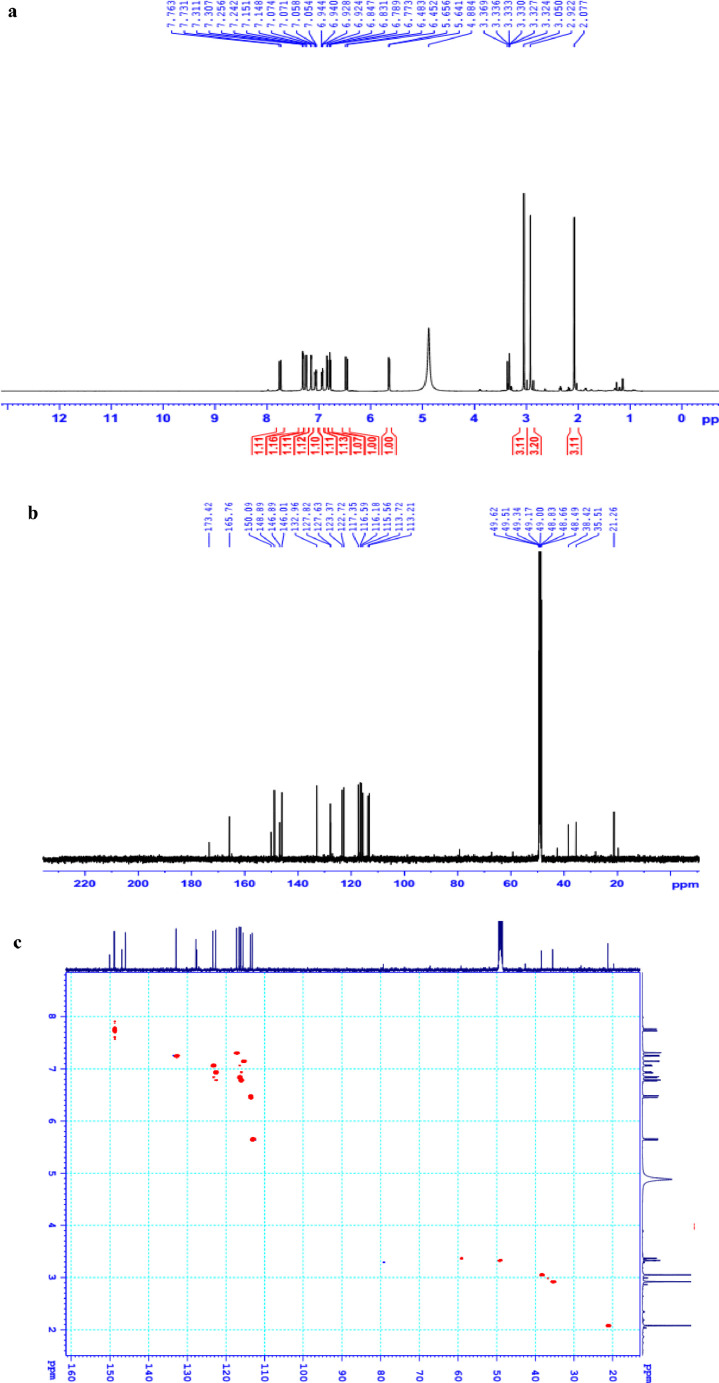

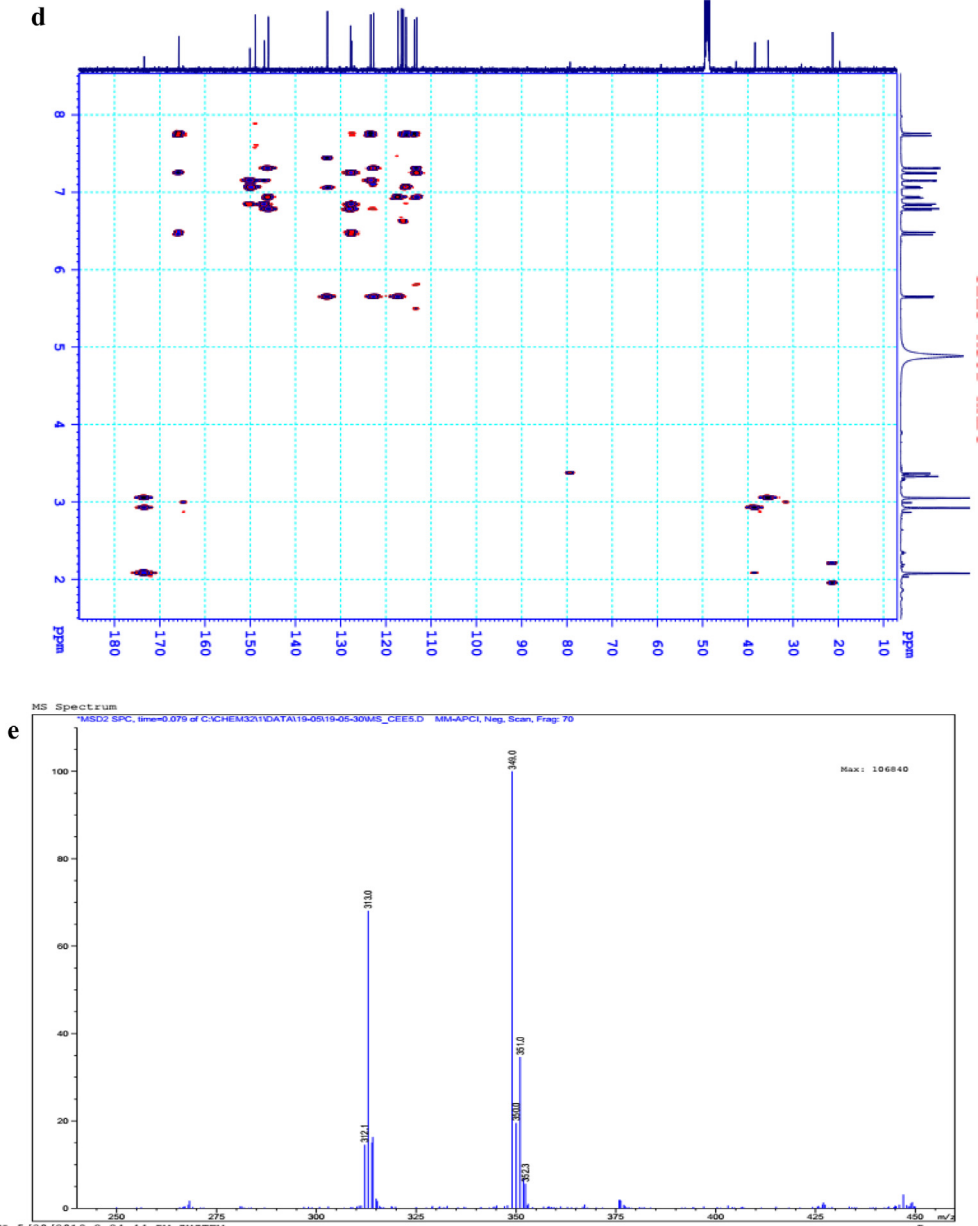
**a**. ^1^H NMR (500 MHz, CD_3_OD) of **3, b**. ^13^C NMR (125 MHz, CD_3_OD) of **3, c**. HSQC of **3, d**. HMBC of **3, e**. (-) APCI-MS of **3**.

**Fig. 5:**
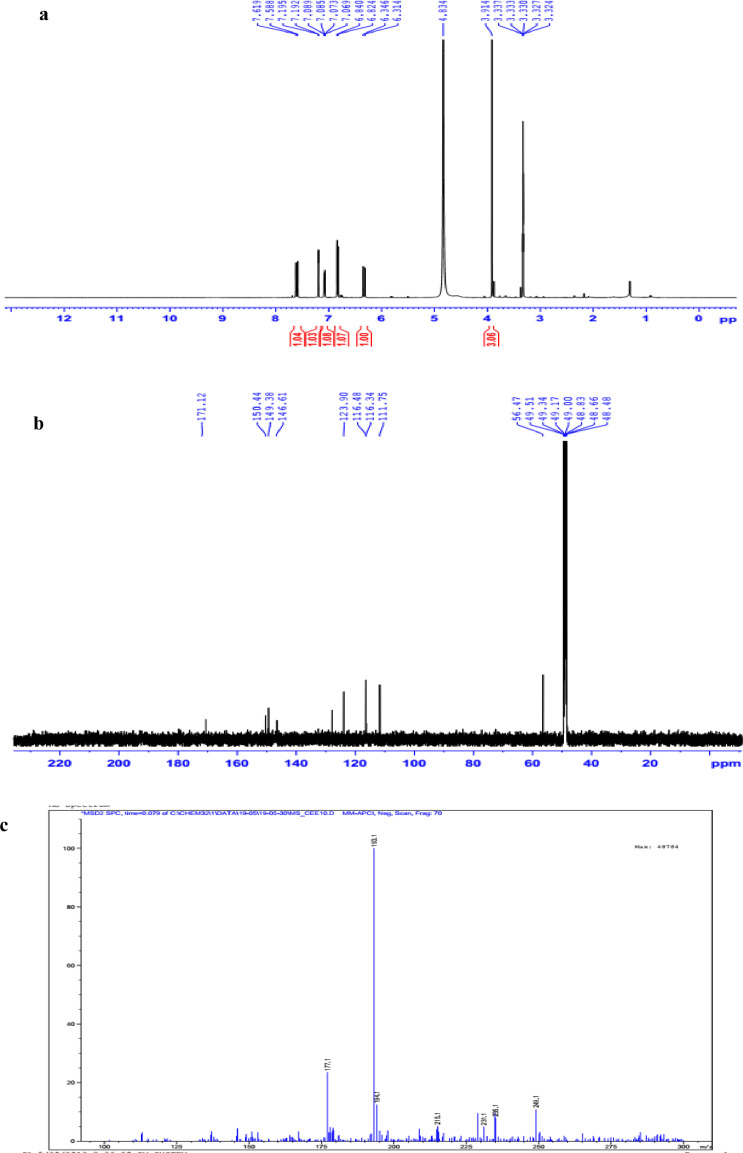
**a**. ^1^H NMR (500 MHz, CD_3_OD) of **4, b**. ^13^C NMR (125 MHz, CD_3_OD) of **4, c**. (-) APCI-MS of **4**. 1D NMR, and APCI-MS of the compound **5** are shown in Fig. 6a-c.

**Fig. 6:**
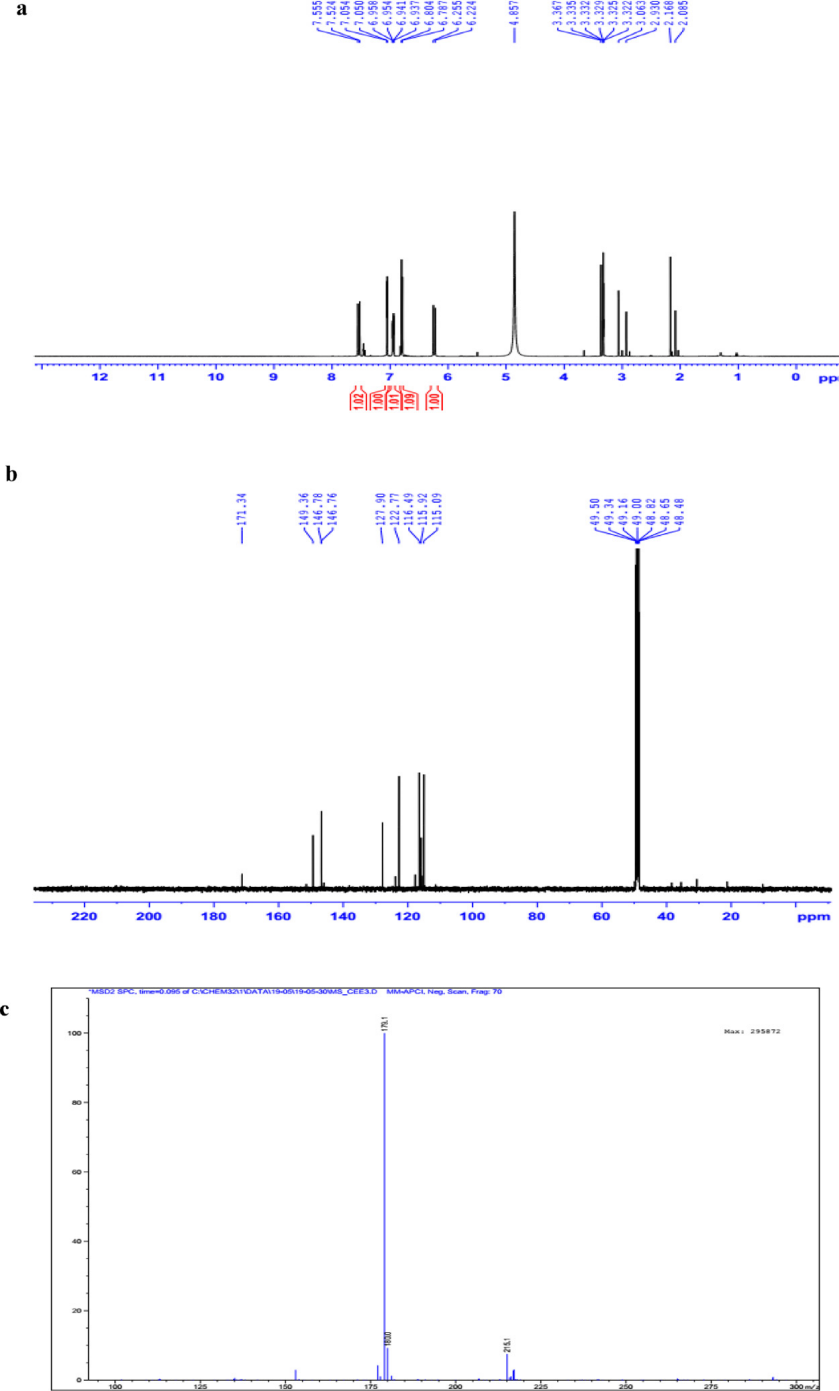
**a**. ^1^H NMR (500 MHz, CD_3_OD) of **5, b**. ^13^C NMR (125 MHz, CD_3_OD) of **5, c**. (-) APCI-MS of **5**.

**Fig. 7:**
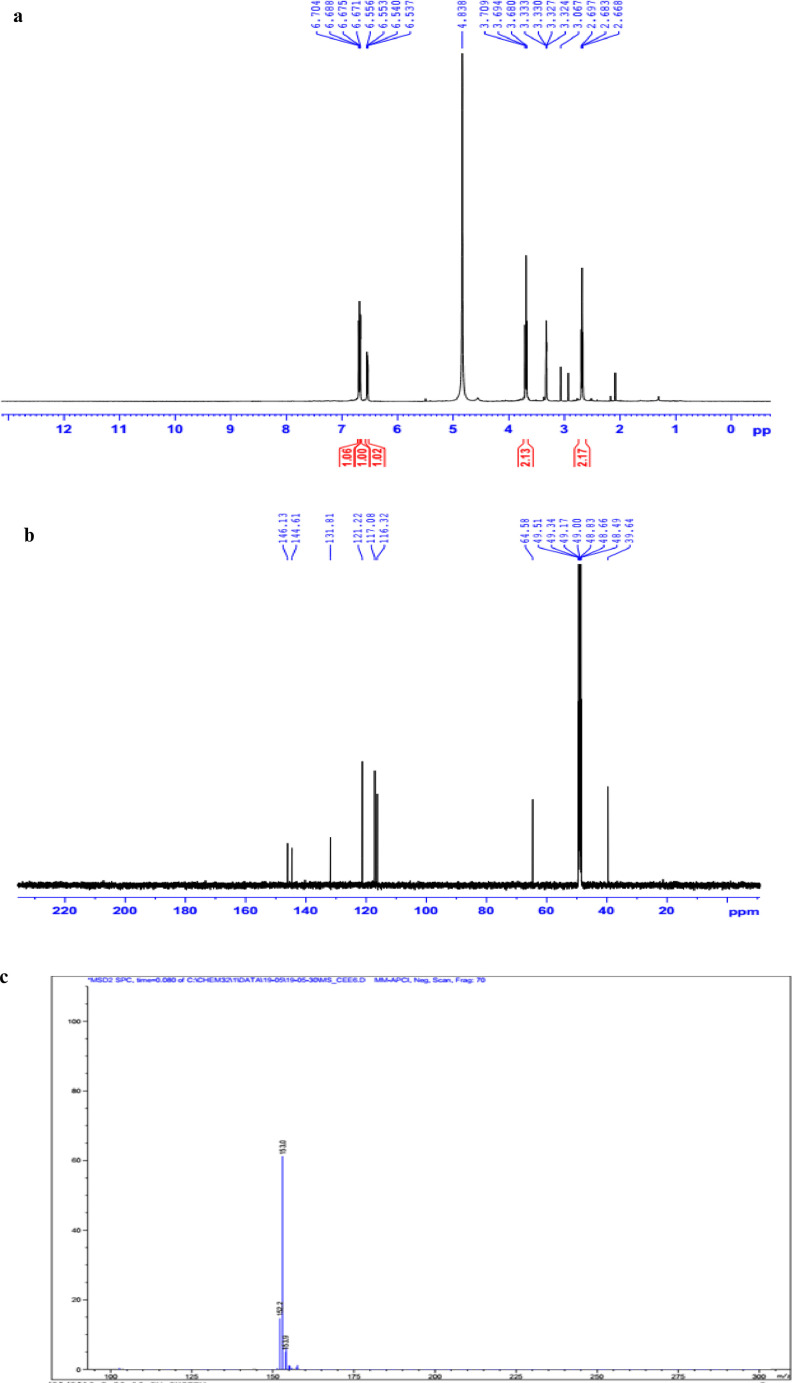
**a**. ^1^H NMR (500 MHz, CD_3_OD) of **6, b**. ^13^C NMR (125 MHz, CD_3_OD) of **6, c**. (-) APCI-MS of **6**.

**Fig. 8:**
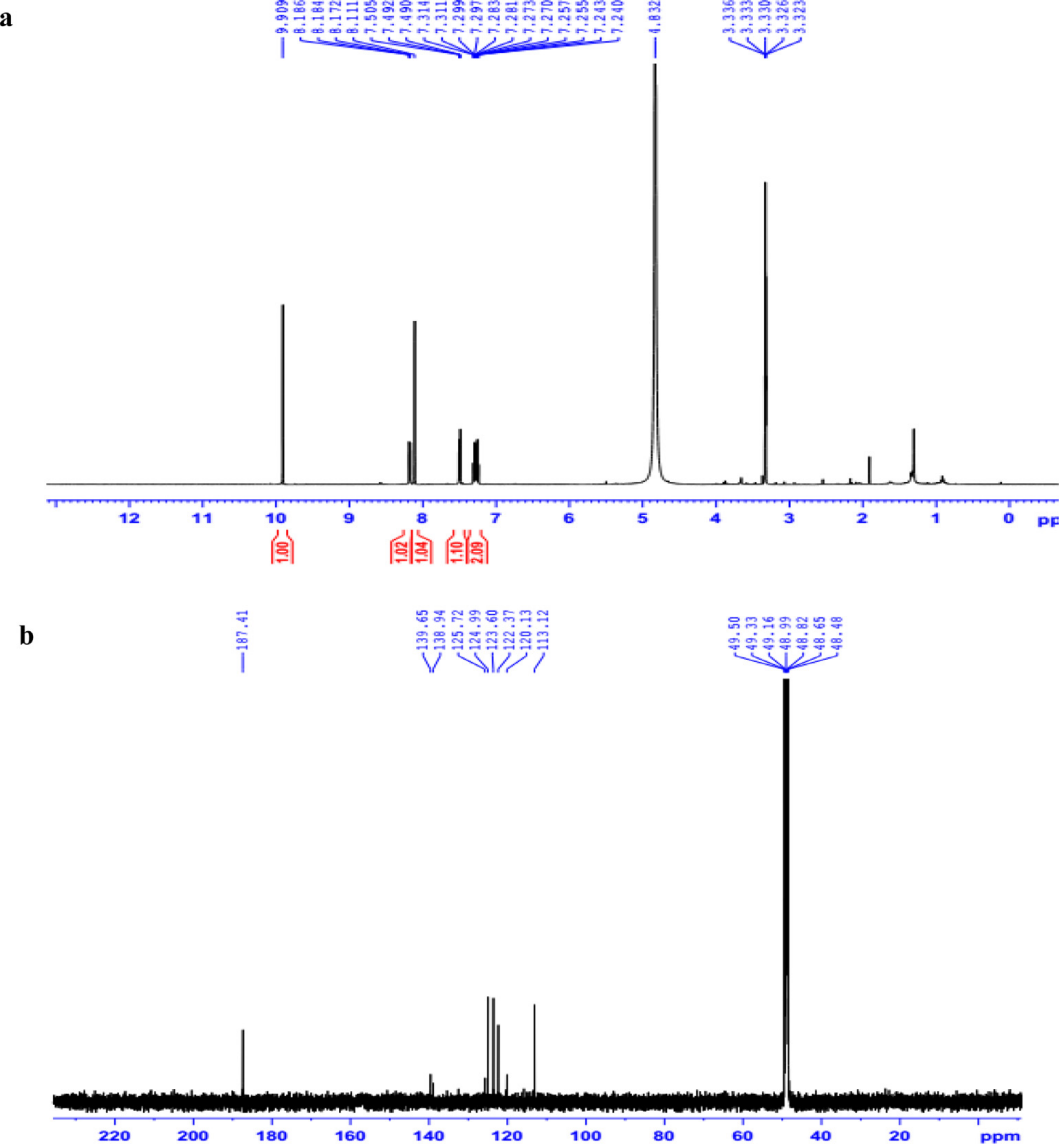

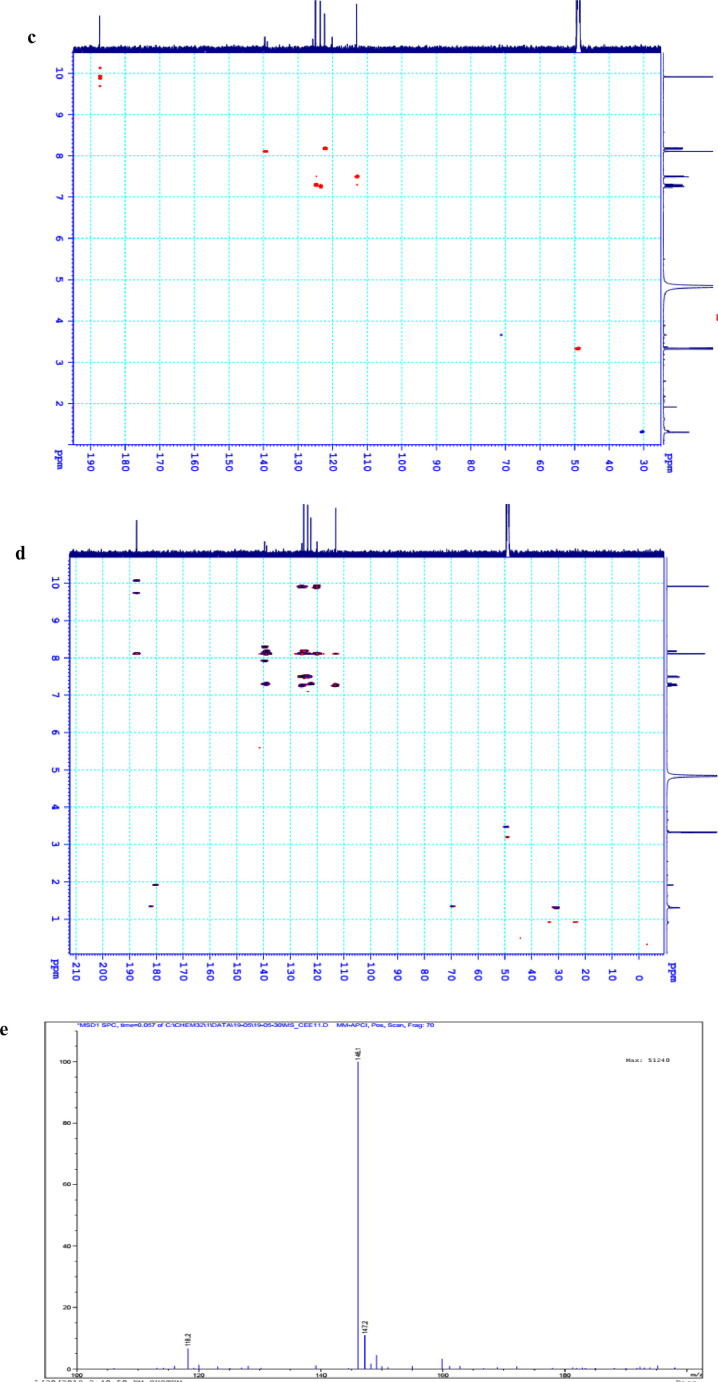
**a**. ^1^H NMR (500 MHz, CD_3_OD) of **7, b**. ^13^C NMR (125 MHz, CD_3_OD) of **7, c**. HSQC of **7, d**. HMBC of **7, e**. (-) APCI-MS of **7**.

The data set presented in this article focus on the antiplatelet aggregation, anticoagulant and antioxidant activity of extracts from *C. edulis* Ker rhizome and its secondary metabolites (Tables 1 and 2). The identification of the isolated compounds from the ethyl acetate fraction of rhizomes of *Canna edulis* described in the research article [1]. In addition, the article also provides the information on the spectroscopic data of seven compounds **1**–**7** isolated from the ethyl acetate fraction of *C. edulis* rhizomes (Fig. 1). The ^1^H NMR of **1**–**7** are shown in Figs. 2a-7a and 8a, respectively. The ^13^C NMR of **1**–**7** are shown in Figs 2b-7b and 8b, respectively. 2D ^1^H–^13^C heteronuclear single quantum coherence (HSQC) of **1, 3** and **7** are shown in Figs. 2c, 4c and 8c, respectively. 2D ^1^H–^13^C heteronuclear multi-bond correlation (HMBC) spectra of **1, 3** and **7** are shown in Figs. 2d, 4d and 8d, respectively. APCI-MS of **1, 3**–**6** and **7** are shown in Figs. 1e, 3e, 4c-6c and 7e, respectively. Analyses of the spectra of **1**–**7** are reported in the research article [1].

**Table 1:** Raw data on antiplatelet aggregation induced by ADP and collagen and anticoagulant activity of *C. edulis* rhizome extracts and its secondary metabolites isolated from the acetyl actate fraction.

Sample	Conc. (mg/mL)		Antiplatelet aggregation activity								
			ADP			Collagen			Anticoagulant activity		
			%I (%)	AUC	Slope	%I	AUC	Slope	PT (s)	APTT (s)	TT (s)
CE.R.Et	4	1st	56.20	131.0	51.0	55.00	131.0	45.0	12.1	25.3	18.8
		2nd	57.30	115.0	55.0	60.00	142.0	40.0	11.3	26.6	17.5
		3rd	52.62	143.0	51.0	62.00	137.0	38.0	11.5	28.3	18.3
	2	1st	24.70	257.0	75.0	30.00	188.0	62.0	10.8	23.8	16.8
		2nd	20.00	250.0	70.0	32.00	170.0	60.0	10.8	23.8	17.0
		3rd	21.67	240.0	76.0	27.00	190.0	56.0	11.3	25.7	17.5
	1	1st	10.00	301.0	86.0	17.00	277.0	89.0	10.9	24.0	16.1
		2nd	15.10	280.0	90.0	15.00	290.0	84.0	10.9	25.3	17.5
		3rd	14.13	292.0	86.0	20.00	283.0	86.0	11.4	28.0	16.9
CE.R.EA	4	1st	93.75	9.0	5.0	98.31	1.2	4.0	45.9	135.8	17.8
		2nd	98.65	7.0	6.0	98.31	0.9	4.0	41.9	104.3	18.8
		3rd	97.10	10.0	6.0	98.21	1.4	7.0	37.8	101.4	18.4
	2	1st	98.39	11.0	5.0	98.65	4.0	6.0	16.1	42.4	16.4
		2nd	98.44	13.0	7.0	98.67	3.8	7.0	15.9	39.1	17.9
		3rd	100.00	11.0	6.0	98.31	2.8	9.0	15.4	42.6	17.5
	1	1st	98.39	14.0	7.0	98.65	8.0	8.0	12.8	30.4	16.5
		2nd	92.19	16.0	6.0	94.67	6.0	7.0	13.6	31.4	17.1
		3rd	100.00	12.0	7.0	98.31	7.0	9.0	12.1	30.6	16.1
CE.R.W	4	1st	52.30	130.0	53.0	15.00	189.0	52.0	11.9	25.3	18.1
		2nd	45.10	145.0	55.0	20.00	181.0	45.0	13.0	29.3	20.5
		3rd	49.00	144.0	57.0	16.95	166.0	49.0	12.1	26.5	18.1
	2	1st	40.10	235.0	70.0	10.00	221.0	60.0	11.6	25.2	17.8
		2nd	32.10	211.0	64.0	13.00	200.0	57.0	12.5	28.5	19.0
		3rd	38.38	219.0	63.0	15.00	192.0	55.0	11.5	26.4	17.4
	1	1st	35.11	234.0	74.0	11.00	299.0	90.0	11.4	22.9	17.3
		2nd	34.40	240.0	68.0	12.70	313.0	94.0	12.4	28.1	18.7
		3rd	25.96	269.0	70.0	12.00	298.0	88.0	11.6	26.3	17.0
Compound 1	0.4	1st	35.90	200.0	61.0	0.00	300.0	94.0	12.0	31.1	17.0
		2nd	30.50	178.0	65.0	0.00	285.0	101.0	12.1	30.8	17.1
		3rd	28.90	184.0	70.0	0.00	307.0	102.0	12.3	31.0	16.9
	0.2	1st	17.91	272.0	81.0	0.00	310.0	99.0	11.0	30.1	16.1
		2nd	16.92	243.0	74.0	0.00	289.0	105.0	11.5	29.8	16.5
		3rd	18.93	250.0	79.0	0.00	315.0	99.0	11.2	30.5	17.0
	0.1	1st	14.93	277.0	79.0	0.00	302.0	90.0	NA	NA	NA
		2nd	15.25	303.0	86.0	0.00	291.0	100.0	NA	NA	NA
		3rd	15.09	281.0	85.0	0.00	307.0	98.0	NA	NA	NA
Compound 2	0.4	1st	28.10	180.0	57.0	25.37	213.8	57.0	26.7	32.1	17.0
		2nd	37.20	169.0	64.0	37.31	197.0	49.0	27.5	31.5	17.2
		3rd	32.30	176.0	59.0	33.1	220.0	60.0	25.9	33.1	17.1
	0.2	1st	12.11	242.0	59.0	14.93	224.5	66.0	11.4	29.9	17.0
		2nd	8.99	260.0	68.0	22.7	242.0	68.0	12.2	32.1	17.1
		3rd	9.57	256.0	75.0	20.0	250.0	59.0	12.5	29.0	17.4
	0.1	1st	5.10	289.0	74.0	7.46	263.0	81.0	11.4	30.1	NA
		2nd	3.20	310.0	80.0	9.10	280.5	90.0	12.2	32.0	NA
		3rd	4.80	315.0	85.0	8.10	282.0	83.0	11.0	31.1	NA
Compound 3	0.1	1st	56.22	154.0	6.0	98.39	0.8	6.0	12.3	32.1	17.2
		2nd	49.53	141.0	7.0	89.74	1.2	6.0	11.4	31.9	17.1
		3rd	55.07	148.0	5.0	100.0	1.0	5.0	12.7	32.5	17.2
	0.05	1st	49.50	176.0	59.0	43.40	188.0	8.0	12.2	31.8	17.4
		2nd	49.10	165.0	64.0	35.90	196.5	6.0	11.8	31.3	17.0
		3rd	40.98	157.0	56.0	38.58	192.0	5.0	12.1	31.1	17.2
	0.025	1st	37.10	179.0	63.0	17.74	205.0	67.0	NA	NA	NA
		2nd	35.80	191.0	68.0	15.91	221.0	71.0	NA	NA	NA
		3rd	28.56	175.0	75.0	22.30	220.0	71.0	NA	NA	NA
Aspirin	0.1	1st	NA	NA	NA	100.0	1.0	6.0			
		2nd	NA	NA	NA	98.21	0.5	6.0			
		3rd	NA	NA	NA	99.12	0.8	7.0			
Ticagrelor	0.002	1st	75.5	13.0	47.0	NA	NA	NA			
		2nd	77.4	8.9	45.0	NA	NA	NA			
		3rd	70.6	14.0	44.0	NA	NA	NA			
DMSO	0.10%	1st	0.00	310.0	88.0	0.00	299.0	108.0	11.6	26.5	16.8
		2nd	0.00	320.0	92.0	0.00	310.0	100.0	12.5	28.2	16.7
		3rd	0.00	315.0	95.0	0.00	311.0	100.0	11.7	28.1	17.5
Heparin	0.2 IU/mL	1st							NA	42.5	29.6
		2nd							NA	45.5	31.5
		3rd							NA	45.8	33.5
	2 IU/mL	1st							26.9	NA	NA
		2nd							27.8	NA	NA
		3rd							29.5	NA	NA

Conc.: concentration, ADP: adenosine diphosphate, CE.R.Et: total ethanol extract of C. edulis rhizome, CE.R.EA: ethyl acetate fraction of C. edulis rhizome, CE.R.W: water fractions of C. edulis rhizome; compound 1: 5-hydroxy-6-methyl-2H-pyran-2-one, compound 2: epimedokoreanone A, compound 3: nepetoidin B, DMSO: dimethylsulfoxide, PT: prothrombin time, APTT: activated partial thromboplastine time, TT: thrombin time, DMSO: dimethylsulfoxide.

**Table 2:** Raw data on antioxidant activity of *C. edulis* rhizome extracts and its secondary metabolites isolated from the acetyl actate fraction.

	Assay		CE.R.Et	CE.R.H	CE.R.EA	CE.R.W	1	2	Trolox	Ascorbic acid
IC50 (mg/mL)	DPPH	1st	2.95	5.75	0.23	8.72	1.97	0.15		0.08
		2nd	3.04	6.13	0.23	3.89	2.10	0.16		0.08
		3rd	3.04	6.11	0.23	5.06	2.17	0.15		0.08
	ABTS	1st	4.29	5.79	0.32	9.8	0.65	0.074	0.092	
		2nd	4.93	6.26	0.49	9.5	0.78	0.07	0.091	
		3rd	4.44	6.03	0.41	9.53	0.69	0.07	0.091	

*CE.R.Et: total ethanol extract of C. edulis rhizome, CE.R.EA: ethyl acetate fraction of C. edulis rhizome, CE.R.W: water fractions of C. edulis rhizome, ABTS: 2,2′-azino-bis-3-ethylbenzthiazoline-6-sulphonic acid, DPPH: 1,1-diphenyl-2 picrylhydrazyl, IC50: the half maximal inhibitory concentration,****1****: 5-hydroxy-6-methyl-2H-pyran-2-one,****2****: epimedokoreanone A*.

NMR information of each compounds are described as below:

*5-Hydroxy-6-methyl-2H-pyran-2-one (1):*

^1^H NMR (500 MHz, CDCl_3_) δ_H_ ppm: 2.36 (3H, s, 6-CH_3_), 6.41 (1H, d, *J* = 5.5 Hz, H-3), 7.69 (1H, d, *J* = 5.5 Hz, H-4).

^13^C NMR (125 MHz, CDCl_3_) δ_C_ ppm: 172.91 (C-2), 112.89 (C-3), 154.25 (C-4), 143.12 (C-5), 148.67 (C-6), 14.26 (6-CH_3_).

APCI-MS positive *m/z*: 127.2 [M + H]^+^

1D NMR, 2D NMR, and APCI-MS of the compound **1** are shown in Fig. 2a-e.

*Epimedokoreanone A (2):*

^1^H NMR (500 MHz, CDCl_3_ + CD_3_OD) δ_H_ ppm: 2.33 (3H, s, 6-CH_3_), 7.80 (1H, s, H-3)

^13^C NMR (125 MHz, CDCl_3_ + CD_3_OD) δ_C_ ppm: 168.76 (C-2), 139.08 (C-3), 144.27 (C-4), 150.60 (C-5), 141.53 (C-6), 13.95 (6-CH_3_).

^1^H and ^13^C NMR of the compound **2** are shown in Fig. 3a and b.

*Nepetoidin B (3):*

^1^H NMR (500 MHz, CD_3_OD) δ_H_ ppm: 7.14 (1H, d, *J* = 1.5 Hz, H-2), 6.83 (1H, d, *J* = 8.0 Hz, H-5), 7.05 (1H, dd, *J* = 1.5, 8.0 Hz, H-6), 7.74 (1H, d, *J* = 15.8 Hz, H-7), 6.46 (1H, d, *J* = 15.8 Hz, H-8), 7.30 (1H, d, *J* = 2.0 Hz, H-2′), 6.77 (1H, d, *J* = 8.0 Hz, H-5′), 6.93 (1H, dd, *J* = 2.0, 8.0 Hz, H-6′), 5.64 (1H, d, *J* = 7.3 Hz, H-7′), 7.24 (d, *J* = 7.3 Hz, H-8′).

^13^C NMR (125 MHz, CD_3_OD) δ_C_ ppm: 127.63 (C-1), 115.56 (C-2), 146.89 (C-3), 150.09 (C-4), 116.59 (C-5), 123.37 (C-6), 148.89 (C-7), 113.73 (C-8), 165.76 (C-9), 127.82 (C-1′), 117.35 (C-2′), 146.01 (C-3′), 146.01 (C-4′), 116.18 (C-5′), 122.73 (C-6′), 113.21 (C-7′), 132.96 (C-8′).

APCI-MS negative *m/z*: 313.0 [M-H]^−^

1D NMR, 2D NMR, and APCI-MS of the compound **3** are shown in Fig. 4a-e.

*Ferulic acid (4):*

^1^H NMR (500 MHz, CD_3_OD) δ_H_ ppm: 7.19 (1H, d, *J* = 2.0 Hz, H-2), 6.82 (1H, d, *J* = 8.0 Hz, H-5), 7.07 (1H, dd, *J* = 2.0, 8.0 Hz, H-6), 6.32 (1H, d, *J* = 16.0 Hz, H-7), 7.58 (1H, d, *J* = 16.0 Hz, H-8), 3.91 (3H, s, 4–OCH_3_).

^13^C NMR (125 MHz, CD_3_OD) δ_C_ ppm: 127.91 (C-1), 111.75 (C-2), 149.38 (C-3), 150.44 (C-4), 116.34 (C-5), 123.48 (C-6), 146.61 (C-7), 116.48 (C-8), 171.12 (C-9), 56.47 (4–OCH_3_).

APCI-MS negative *m/z*: 193.1 [M-H]^−^

1D NMR, and APCI-MS of the compound **4** are shown in Fig. 5a-c.

*Caffeic acid (5):*

^1^H NMR (500 MHz, CD_3_OD) δ_H_ ppm: 7.05 (1H, d, *J* = 2.0 Hz, H-2), 6.79 (1H, dd, *J* = 8.5 Hz, H-5), 6.94 (1H, d, *J* = 2.0, 8.5 Hz, H-6), 6.22 (1H, d, *J* = 15.5 Hz, H-7), 7.55 (1H, d, *J* = 15.5 Hz, H-8).

^13^C NMR (125 MHz, CD_3_OD) δ_C_ ppm: 127.90 (C-1), 115.09 (C-2), 146.76 (C-3), 149.36 (C-4), 122.77 (C-5), 116.49 (C-6), 146.78 (C-7), 115.92 (C-8), 171.34 (C-9.

APCI-MS negative *m/z*: 179.1 [M-H]^−^ .

*Hydroxytyrosol (6):*

^1^H NMR (500 MHz, CD_3_OD) δ_H_ ppm: 6.67 (1H, d, *J* = 2.0 Hz, H-2), 6.69 (1H, dd, *J* = 8.0 Hz, H-5), 6.54 (1H, d, *J* = 2.0, 8.0 Hz, H-6), 3.69 (2H, t, *J* = 7.5 Hz, H-7), 2.68 (2H, t, *J* = 7.5 Hz, H-8).

^13^C NMR (125 MHz, CD_3_OD) δ_C_ ppm: 131.81 (C-1), 116.32 (C-2), 144.61 (C-3), 146.13 (C-4), 117.08 (C-5), 121.22 (C-6), 39.64 (C-7), 64.58 (C-8).

APCI-MS negative *m/z*: 153.0 [M-H]^−^ .

1D NMR, and APCI-MS of the compound **6** are shown in Fig. 6a-c.

*1H-indole-3-carboxaldehyde (7):*

^1^H NMR (500 MHz, CD_3_OD) δ_H_ ppm: 7.24 (1H, dt, *J* = 1.5, 7.5 Hz, H-5), 7.29 (1H, dt, *J* = 1.5, 7.5 Hz, H-6), 7.49 (1H, br.d, *J* = 7.5 Hz, H-7), 8.11 (1H, s, H-2), 8.18 (1H, br.d, *J* = 7.5 Hz, H-4).

^13^C NMR (125 MHz, CD_3_OD) δ_C_ ppm: 113.12 (C-7), 120.13 (C-3), 122.37 (C-4), 123.60 (C-5), 124.99 (C-6), 125.72 (C-9), 138.94 (C-8), 139.65 (C-2), 187.41 (C-10).

APCI-MS positive *m/z*: 146.1 [M + H]^+^.

1D NMR, 2D NMR and APCI-MS of the compound **7** are shown in Fig. 8a-e.

## 2 Experimental design, materials, and methods

### 2.1 Collection of plant

*C. edulis* Ker rhizome*,* identified by the plant researcher Thi Thanh Huong Le – Thai Nguyen University of Sciences, Thai Nguyen, Vietnam, was collected in Thai Nguyen province, Vietnam. A voucher specimen number CE.R.TN01 is deposited at Department of Life Sciences, University of Science and Technology of Hanoi, Vietnam Academy of Science and Technology.

### 2.2 Extraction and isolation of compounds

The dry powder of *C. edulis* rhizome (CE.R) (6.2 kg) was macerated in ethanol 96% at room temperature and the solvent was evaporated. The total ethanol extract (CE.R.Et) then was fractionated with *n*-hexane, ethyl acetate (EA) and water. The *n*-hexane extract (CE.R.H, 13.7 g), EA extract (CE.R.EA, 20.0 g) and the aqueous extracts (CE.R.W, 315 g) were obtained and evaporated under vacuum and then stored at 4–6 °C for further use.

The isolation of compounds from CE.R.EA was reported in the research article [1]. NMR spectra were acquired using a Bruker Avance 500 MHz instrument using TMS as internal standard (500 MHz for ^1^H, 125 MHz for ^13^C). APCI-MS was carried out using a AGILENT 6120 mass spectrometer at the Institute of Marine Biochemistry, Vietnam Academy of Science and Technology.

### 2.3 Antiplatelet aggregation activity test

Blood from healthy volunteers aged 18 - 35, who did not take any drugs for 3 weeks and were fasting overnight, was collected. A complete blood count was measured before platelet aggregation and coagulation experiments to ensure they had normal blood cell counts. The venous blood taken was put into a 3.2% sodium citrate tube and then centrifuged at 500 rpm for 10 min to take platelet-rich plasma (PRP). Blood sample was also centrifuged at 3000 rpm for 10 min to take platelet-poor plasma (PPP). Platelets were counted under microscope, and the platelet count was adjusted to 250 ± 25 × 10^9^/L in PRP.

The platelet aggregation was done using the turbidimetric method [2] in triplicate. Briefly, 450 µL of PRP and 50 µL of extracts or compounds were incubated at 37 °C for 3 min. Platelet aggregation was stimulated by ADP 10 µM or collagen 2 µg/mL. DMSO 0.1% and aspirin 0.1 mg/mL in the case of collagen or ticagrelor 0.002 mg/mL in the case of ADP were used as negative and positive control, respectively. Amplitude-time curves over 6 min were recorded and then three parameters: the maximum aggregation, the area under the platelet aggregation curve (AUC) and the maximum slope of the aggregation curve were collected. The percentage inhibition of platelet aggregation (%*I*) was calculated as %*I* = $\frac{X-Y}{X}$ x 100%, *X* is maximum percentage aggregation of the negative control; *Y* is maximum aggregation percentage of the sample [1].

### 2.4 Anticoagulant activity test

First, 450 μL of PPP were mixed with 50 μL of plant extracts or pure compounds and the mixtures then were incubated at 37° for 5 min. Then, PT, APTT and TT were measured using Sysmex CS-2100i machine (Japan) following the previously described method [3] to assess the anticoagulant activity. Heparin 0.2 IU/mL (for APTT and TT assay) or 2 IU/mL (for PT assay) and DMSO 0.1% were used as the positive and negative control, respectively. The experiment was done in triplicate.

### 2.5 Antioxidant activity test

#### 2.5.1 DPPH assay

The DPPH assay was carried out in triplicate according to the previously described method with some modifications [4]. The extracts and compounds were dissolved in DMSO 100% and then diluted into tested concentrations. Then, 190 µL DPPH (0.1 mM) dissolved in methanol was incubated with 10 µL sample at 37 °C for 20 min. The absorbance was read at 517 nm. Ascorbic acid dissolved in distill water at 10, 25 and 50 µg/mL was used as the positive control. The percentage inhibition of free radicals was calculated as% Inhibition = 100 – ($\frac{OD_{s}}{OD_{c}}\;$x100%), *OD_s_* is an average optical density of the sample, *OD_c_* is an average optical density of the control. The sample concentrations needed for 50% inhibition of the radicals (IC_50_ values) were calculated.

#### 2.5.2 ABTS assay

The working solution ABTS^+^ was prepared by mixing 7 mM ABTS solution with 2.45 mM potassium persulfate solution (ratio 1:1) and kept in the dark for 14–16 h at room temperature [5]. This mixture was further diluted in ethanol to reach the absorbance of 0.7 ± 0.02 at 734 nm. Then, 10 µL samples dissolved in ethanol at different concentrations were incubated with 190 µL of the diluted ABTS^+^ solution at room temperature for 10 min. The absorbance was read at 734 nm using a Microplate spectrophotometer. Trolox at 50, 100, 200, 500 µg/mL were used as the positive control. IC_50_ values were also calculated as described above. The experiment was done in triplicate.

## Ethics statement

The study was carried out in accordance with Declaration of Helsinki for experiments involving humans. Informed consent was obtained for experimentation with human subjects.

## Declaration of Competing Interest

The authors declare that they have no known competing financial interests or personal relationships which have, or could be perceived to have, influenced the work reported in this article.
